# Effectiveness of Extended Reality‐Based Cardiopulmonary Resuscitation Training for Healthcare Students: A Protocol for a Systematic Review and Meta‐Analysis

**DOI:** 10.1002/nop2.70721

**Published:** 2026-07-29

**Authors:** John Raymund P. Suazo, Grasiela Piuvezam, Isac Davidson S. F. Pimenta, Lucía López Ferrándiz, Manuel Pardo Ríos, Rafael Castro‐Delgado

**Affiliations:** ^1^ Department of Medicine University of Oviedo Oviedo Spain; ^2^ Department of Global Public Health Karolinska Institute Stockholm Sweden; ^3^ Department of Public Health Federal University of Rio Grande do Norte Natal Brazil; ^4^ Post‐Graduation Program in Public Health Federal University of Rio Grande do Norte Natal Brazil; ^5^ Systematic Review and Meta‐Analysis Laboratory Federal University of Rio Grande do Norte (LABSYS‐UFRN/CNPQ) Natal Brazil; ^6^ Health Sciences UCAM Universidad Católica de Murcia Murcia Spain; ^7^ Health Service of the Principality of Asturias (SAMU‐Asturias), Research Group on Prehospital Care and Disasters (GIAPREDE) Health Research Institute of the Principality of Asturias Oviedo Spain

**Keywords:** cardiopulmonary resuscitation, extended reality, healthcare education, nursing

## Abstract

**Introduction:**

Sudden cardiac arrest remains a major cause of global mortality, underscoring the importance of cardiopulmonary resuscitation. Extended reality (XR) technologies, including virtual, augmented, and mixed reality, have recently emerged as innovative approaches to enhance simulation‐based CPR education for nursing and healthcare students.

**Aim:**

To present the protocol for a systematic review and meta‐analysis intended to evaluate the effects of XR‐based interventions on CPR training outcomes among healthcare students.

**Design and Reporting Method:**

The protocol was developed following the Preferred Reporting Items for Systematic Review and Meta‐Analysis Protocols (PRISMA‐P) recommendations.

**Data Sources:**

Electronic database searches will be performed in PubMed/MEDLINE, EMBASE, CINAHL, Cochrane Library, Web of Science, and Scopus.

**Methods and Analysis:**

Relevant studies will be searched through systematic database and reference screening. Randomised controlled trials and quasi‐experimental studies investigating XR‐supported CPR training will be considered for inclusion. Two reviewers will independently conduct study screening and data extraction, with disagreements resolved through consultation with a third reviewer. Methodological quality will be evaluated using the Cochrane ROB 2 tool and the ROBINS‐I assessment tool where appropriate. Findings will first be summarised narratively, while meta‐analysis will be undertaken if sufficient methodological homogeneity among studies is observed.

**Implications and Impact:**

Current evidence on XR‐based CPR training has primarily focused on laypersons or practicing professionals, leaving evidence specific to nursing and other healthcare students limited. This review will try to systematically summarise and appraise available studies in this population, with particular attention to reported outcomes related to CPR competencies. The results may help identify evidence gaps and guide future research directions. If XR demonstrates potential advantages, this review may support educators in considering the role of XR technologies in CPR training programs.

**Patient or Public Contribution:**

No public or patient contribution.

**Protocol Registration:**

This protocol is registered in the International Prospective Register of Systematic Reviews (PROSPERO registration number: CRD42024528709).

## 1 Introduction

Sudden cardiac arrest (SCA) remains a major public health concern worldwide and continues to contribute substantially to global mortality (Al‐Shaqsi 2010). In North America and Western Europe alone, SCA has been estimated to account for nearly one‐fifth of all deaths (Al‐Shaqsi 2010). Early recognition of cardiac arrest and prompt initiation of cardiopulmonary resuscitation (CPR) are strongly associated with improved survival outcomes in both out‐of‐hospital and in‐hospital settings (Drummond et al. 2017). Consequently, international organizations such as the American Heart Association and the European Resuscitation Council continue to advocate for broad and effective CPR education initiatives (Cheng et al. 2018; Baldi et al. 2020). Despite widespread implementation of Basic Life Support (BLS) and Advanced Cardiac Life Support (ACLS) training programs, overall survival rates following cardiac arrest remain low, with successful resuscitation reported in only a limited proportion of cases (Jaskiewicz et al. 2020; Gräsner et al. 2021). These findings suggest that ongoing improvements in CPR education and training strategies remain necessary.

Healthcare professionals are expected to respond rapidly and effectively during cardiac arrest events. Nurses, in particular, frequently serve as first‐line responders during in‐hospital resuscitations, are often responsible for initiating CPR while participating in coordinated team‐based care (Moon and Hyun 2019). Nevertheless, competency in BLS cannot be presumed solely on the basis of professional or academic background. Evidence from multiple countries has identified deficiencies in CPR‐related knowledge and psychomotor performance among healthcare students (Willmore et al. 2019; Contri et al. 2017). In some educational settings, inadequate exposure to structured BLS instruction has been associated with insufficient CPR competency among students (Baldi et al. 2020). Furthermore, several studies have demonstrated that learners may struggle to retain CPR‐related knowledge and practical skills over time despite previous training exposure (Baldi et al. 2019). Conventional CPR instruction commonly relies on instructor‐led demonstrations and manikin‐based simulation. Although these approaches remain widely used, they may inadequately reproduce the stress, complexity, and dynamic environment of actual resuscitation events, potentially limiting learner preparedness and confidence during real clinical encounters (Barsom et al. 2020).

Extended reality (XR) technologies, particularly virtual reality (VR) and augmented reality (AR), have increasingly been incorporated into nursing and healthcare education. The adoption of simulation‐based learning has increased substantially since 2014, with a marked acceleration following the COVID‐19 pandemic and growing use of advanced modalities such as immersive VR (Mun et al. 2025). XR‐based simulations offer interactive and immersive learning environments that may complement traditional teaching methods by enabling repeated, standardised practice and addressing challenges related to scalability and resource constraints (Mun et al. 2025; Ota et al. 2024).

Randomised controlled trials have reported that VR‐based CPR training can be an engaging and scalable educational tool, with some evidence of improved psychomotor performance and learner satisfaction compared with conventional training (Bodur et al. 2025). Beyond technical skill acquisition, exposure to unfamiliar, high‐fidelity environments—such as mixed reality settings—has been shown to influence learners' confidence and stress responses, potentially enhancing preparedness for high‐pressure clinical situations (Rushton et al. 2020).

Several systematic reviews have explored XR in CPR training; however, most have focused on laypersons or healthcare professionals broadly, without distinguishing outcomes for undergraduate healthcare students. Additionally, prior reviews have varied in the outcomes assessed, often emphasizing technical skills while omitting non‐technical skills such as confidence and decision‐making. There is currently limited synthesis that specifically evaluates both technical and non‐technical CPR competencies among nursing and other healthcare students.

Given the expanding interest in XR‐assisted learning, a focused synthesis of evidence involving nursing students, who are often the first to initiate in‐hospital resuscitation, is warranted. This review will systematically summarise and appraise available studies in this population, with particular attention to reported outcomes related to CPR competencies. The findings may help identify evidence gaps and inform future research directions. Where XR demonstrates potential advantages, this review may support educators in considering its role in CPR training programs.

### 1.1 Aims

This study aims to develop a systematic review and meta‐analysis protocol examining the effects of extended reality technologies on CPR training outcomes among undergraduate healthcare students.

## 2 Methodology

### 2.1 Design

The present study outlines the protocol for a systematic review and meta‐analysis examining the use of extended reality (XR) technologies in cardiopulmonary resuscitation (CPR) education among healthcare students. Reporting of the protocol was developed in accordance with the Preferred Reporting Items for Systematic Review and Meta‐Analysis Protocols (PRISMA‐P) recommendations (Moher et al. 2015). To promote methodological transparency, the protocol has been prospectively registered in the International Prospective Register of Systematic Reviews (PROSPERO registration number: CRD42024528709).

### 2.2 Search Strategy

A comprehensive literature search will be undertaken using the following databases: PubMed/MEDLINE, EMBASE, CINAHL, Cochrane Library, Web of Science, and Scopus. Search terms will combine controlled vocabulary and free‐text keywords associated with CPR, virtual reality, augmented reality, mixed reality, and healthcare students. Boolean operators, truncation symbols, and database‐specific indexing terms will be incorporated where appropriate to optimise the search strategy. The complete PubMed/MEDLINE search strategy is presented in Table 1. Search syntax will subsequently be modified according to the indexing systems of each database. In addition to database searching, the reference lists of eligible studies will be screened to identify potentially relevant articles not captured during the primary search process.

**TABLE 1:** Sample search strategy for PubMed/MEDLINE.

	PubMed/MEDLINE search strategy
#1	(‘Virtual Reality’[Mesh]) OR (Reality, Virtual) OR (Virtual Reality, Educational) OR (Educational Virtual Realit*) OR (Reality, Educational Virtual) OR (Virtual Realities, Educational) OR (Virtual Reality, Instructional) OR (Instructional Virtual Realit*) OR (Instructional Virtual Reality) OR (Realities, Instructional Virtual) OR (Reality, Instructional Virtual) OR (Virtual Realities, Instructional)
#2	(‘Augmented Reality’[Mesh]) OR (Augmented Realit*) OR (Realities, Augmented) OR (Reality, Augmented) OR (Mixed Realit*) OR (Realities, Mixed) OR (Reality, Mixed)
#3	#1 OR #2
#4	(‘Cardiopulmonary Resuscitation’[Mesh]) OR (Resuscitation, Cardiopulmonary) OR (CPR) OR (Cardio‐Pulmonary Resuscitation) OR (Cardio Pulmonary Resuscitation) OR (Resuscitation, Cardio‐Pulmonary) OR (Code Blue) OR (Mouth‐to‐Mouth Resuscitation) OR (Mouth to Mouth Resuscitation) OR (Mouth‐to‐Mouth Resuscitations) OR (Resuscitation, Mouth‐to‐Mouth) OR (Resuscitations, Mouth‐to‐Mouth) OR (Basic Cardiac Life Support) OR (Life Support, Basic Cardiac)
#5	#3 AND #4

### 2.3 Eligibility Criteria

#### 2.3.1 Population

Eligible studies will involve undergraduate healthcare students enrolled in disciplines such as nursing, medicine, midwifery, and related health professions. Studies focusing exclusively on laypersons, licensed healthcare professionals, or postgraduate students will not be considered for inclusion.

#### 2.3.2 Intervention

Studies evaluating XR‐supported CPR education or simulation activities will be eligible. XR modalities may include virtual reality (VR), augmented reality (AR), or mixed reality (MR). Studies that do not incorporate XR technologies as part of the CPR training intervention will be excluded.

#### 2.3.3 Comparator

Included studies must compare XR‐based CPR training approaches with conventional or standard educational strategies.

#### 2.3.4 Outcomes

The primary outcomes of interest will include measures related to CPR performance quality and CPR knowledge acquisition. CPR quality indicators may include chest compression depth, compression rate, chest compression fraction, and no excessive ventilation. Secondary outcomes will include both technical and non‐technical competencies, such as retention of psychomotor skills, confidence, communication, teamwork, leadership, and clinical decision‐making. Outcomes must be quantifiable for meta‐analysis (e.g., mean chest compression depth in mm, percentage of correct compressions).

#### 2.3.5 Study Design

Randomised controlled trials and quasi‐experimental studies will be eligible for inclusion. Observational studies, conference abstracts without sufficient data, and case reports will be excluded.

#### 2.3.6 Additional Criteria

No limitations will be imposed regarding publication year, language, publication status, or geographic setting. Table S1 presents the PICO framework and operational definitions applied to the review outcomes.

### 2.4 Search Outcome

**FIGURE 1:**
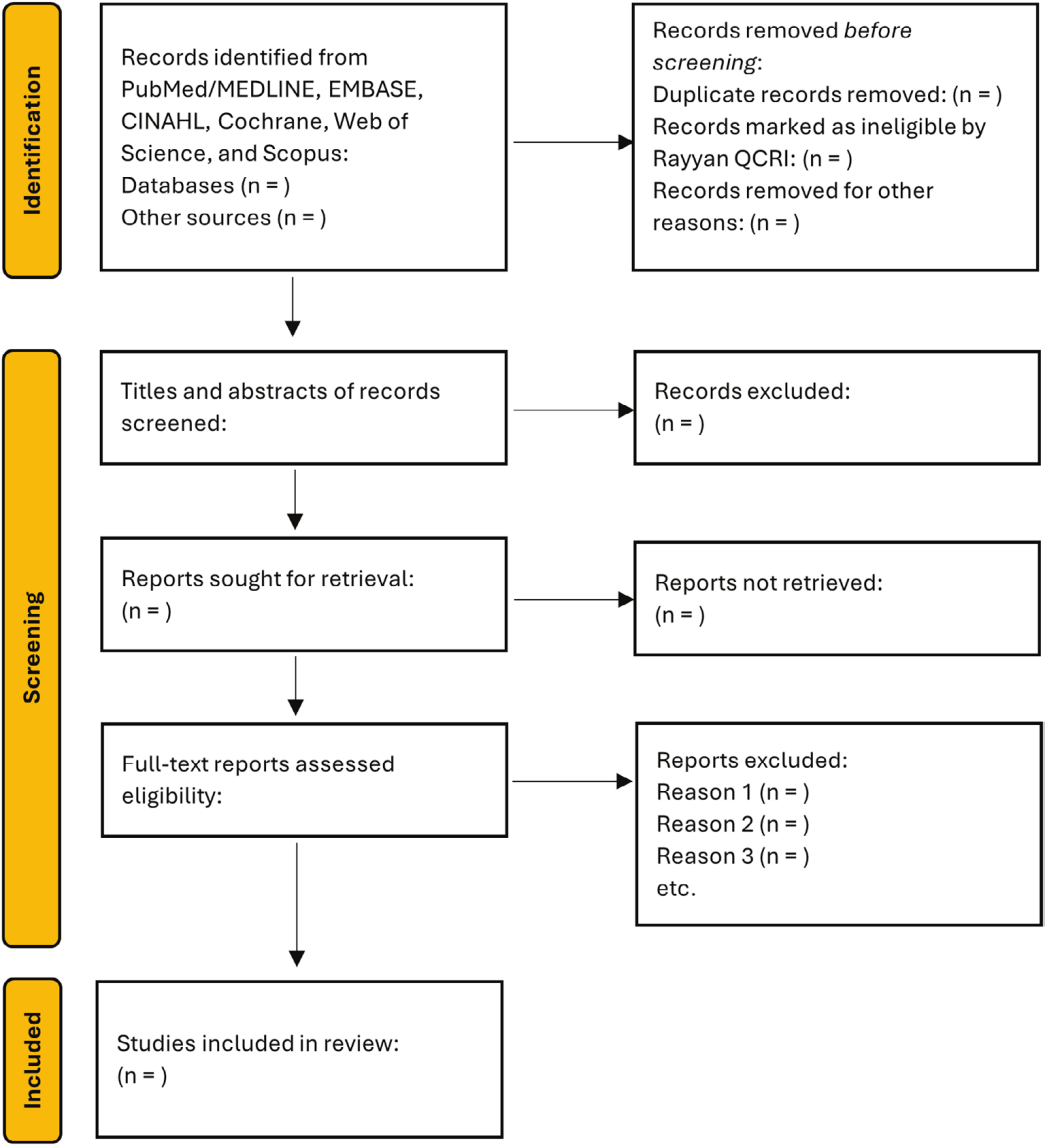
PRISMA‐P guided flow diagram illustrating the study selection process for the systematic review.

Following database retrieval, all identified studies will be imported into Rayyan QCRI to facilitate screening and organisation of records. Duplicate entries will first be identified and removed prior to screening. Two reviewers will independently evaluate titles and abstracts according to the predefined eligibility criteria. Articles considered potentially relevant will subsequently undergo full‐text assessment by the same reviewers. Any disagreements during the screening or eligibility assessment stages will be resolved through discussion, with consultation from a third reviewer when necessary. The overall study selection procedure is illustrated in the PRISMA flow diagram shown in Figure 1.

### 2.5 Quality Appraisal

Methodological quality and risk of bias of the included studies will be evaluated using validated appraisal instruments. Randomised controlled trials will be assessed using the Cochrane Risk of Bias 2 (ROB 2) tool, whereas non‐randomised and quasi‐experimental studies with comparator groups will be appraised using the ROBINS‐I framework. Each study will subsequently be classified according to the corresponding risk‐of‐bias categories, such as low risk, some concerns, or high risk of bias.

### 2.6 Data Abstraction

Two members of the research team will independently perform data extraction using a modified Cochrane data extraction template (Li et al. 2023). Following study selection, extracted information will be compared with AI‐assisted extraction outputs to examine the consistency of the data collected.

Extracted variables will include study details (e.g., title, authors, publication year, journal, and country of origin), participant characteristics (including sex, average age, academic discipline, and level of training), intervention‐related information (type of XR modality, simulation format, and duration of training), and reported outcomes such as CPR quality, knowledge acquisition, skills retention, and learner perceptions. Where important information is incomplete or unclear, attempts will be made to contact the corresponding authors for clarification. If the requested data remain unavailable, the affected information will not be incorporated into the final analysis, and this will be acknowledged as a study limitation.

### 2.7 Synthesis

Findings will initially be summarised through a narrative synthesis describing the main characteristics, interventions, and outcomes reported across the included studies. Where sufficient methodological homogeneity exists, a quantitative synthesis through meta‐analysis will also be undertaken. A random‐effects model will be applied to account for expected variability among studies, including differences in the type of XR used, training design, and populations. Statistical analyses will be performed using Review Manager (RevMan) version 5.4.

For dichotomous variables, data will be reported as risk ratios with corresponding 95% confidence intervals. Continuous outcomes will be analysed using mean differences and 95% confidence intervals. Statistical significance will also be considered in the interpretation of findings. CPR quality will be assessed using quantifiable measures, including, but not limited to, mean chest compression depth (mm) and the percentage of correct compressions. Outcomes will be analysed by timing, with immediate post‐intervention data used where available and the longest follow‐up (e.g., ≥ 3 months) used to assess skills retention.

Variability across studies will be examined using the chi‐square (*χ* ^2^) test and quantified using the I2 statistic. I2 values of approximately 0%, 50%, and 75% will be interpreted as representing low, moderate, and substantial heterogeneity, respectively. If an adequate number of studies are available, publication bias will be explored through funnel plot inspection and assessment of funnel plot asymmetry using regression‐based methods.

Subgroup analyses may be conducted to investigate potential sources of heterogeneity, particularly according to XR modality or learner population. Additional subgroup analyses may be performed based on learner type or training context, subject to data availability. To avoid double‐counting in multi‐arm studies, intervention groups will be combined or shared control groups proportionally split in accordance with the Cochrane Handbook.

The certainty of evidence for each outcome will be evaluated using the Grading of Recommendations Assessment, Development and Evaluation (GRADE) framework.

## 3 Discussion

This study presents the protocol for a systematic review and meta‐analysis investigating the application of extended reality (XR) technologies in cardiopulmonary resuscitation (CPR) training. As technological advancements continue to emerge, there is growing interest in their potential role in supporting the acquisition of lifesaving competencies within healthcare education. However, a systematic evaluation of existing evidence is necessary to better understand how these technologies have been applied and what outcomes have been reported.

The use of immersive technology in training and education offers customisable and repeatable learning, which may be relevant for mastering basic healthcare competencies such as CPR. Extended reality technology often incorporates real‐time feedback and simulated clinical scenarios, which aligns with findings that such technologies have been explored in relation to clinical judgement and decision‐making of healthcare students following clinical simulations (Aebersold and Gonzalez 2024; Yang et al. 2024). Some studies suggest associations with outcomes such as confidence and willingness to perform CPR (Moll‐Khosrawi et al. 2022). Other studies report improvements in learning outcomes, performance, and teamwork during resuscitation (Perron et al. 2021).

While simulation is an integral part of CPR training, traditional methods involving manikins may present logistical challenges related to cost, access, and scheduling, particularly considering the variability in skill retention over time (Drummond et al. 2017). XR technologies have been proposed as a complementary training modality; however, their effectiveness and feasibility require careful evaluation through evidence synthesis (Drummond et al. 2017).

This protocol was developed in accordance with established PRISMA recommendations and incorporates recognised tools for methodological quality appraisal. By conducting searches across multiple databases without restrictions on publication year or geographic setting, this systematic review aims to provide a broad and relevant synthesis of current evidence on XR‐assisted CPR training among healthcare students.

Several potential limitations should nevertheless be considered. Variability in XR modalities, instructional approaches, and reported outcome measures may result in substantial heterogeneity among studies, potentially affecting data comparability. In addition, although efforts will be made to include studies published in different countries, language‐related limitations may still contribute to selection bias. The exclusion of grey literature may likewise reduce the comprehensiveness of the evidence base and increase the possibility of publication bias.

## 4 Conclusion

This review seeks to comprehensively evaluate published evidence regarding the integration of extended reality (XR) technologies into cardiopulmonary resuscitation (CPR) training for healthcare students. By examining both technical and non‐technical training outcomes, the review intends to identify areas where current evidence remains limited or inconsistent. The synthesis of available findings may provide direction for future investigations and support ongoing discussions regarding the incorporation of XR‐based approaches within CPR education programs.

## Author Contributions

J.R.P.S., M.P.R., L.L.F. and I.D.S.F.P. developed the initial conceptualisation of the study. J.R.P.S. wrote the initial draft of the manuscript, while I.D.P. developed the search strategy. J.R.P.S., I.D.S.F.P., G.P. and M.P.R. formulated the methodology and data curation. J.R.P.S. and I.D.S.F.P. will conduct the study selection and investigation, under the oversight and supervision of G.P., M.P.R., and R.C.‐D. J.R.P.S., I.D.S.F.P., G.P. and L.L.F. critically revised this manuscript. Additionally, all authors have engaged in reading, reviewing, and granting their approval for the final protocol, ensuring a cohesive and unified approach to the study.

## Funding

John Raymund P. Suazo was supported by a European Education and Culture Executive Agency (EACEA) scholarship for the Erasmus Mundus Master in Public Health in Disasters, which also covered the article processing charges for this publication.

## Disclosure

*Dissemination*: This study also aims to disseminate the review findings through publication in a peer‐reviewed journal. Any changes to this protocol will be promptly reflected in the PROSPERO registration record, with a detailed explanation of the changes provided in the final report of the review.

## Ethics Statement

This study examines previously published studies that do not contain personally identifiable information about participants, rendering ethical approval unnecessary from a research committee.

## Conflicts of Interest

The authors declare no conflicts of interest.

## Supporting information


**Table S1:** Eligibility criteria for the systematic review.

## Data Availability

As this study is a protocol for a systematic review and meta‐analysis, no primary data were generated or analysed. All data to be used in the review will be derived from published studies and publicly available sources. Any data extracted and analysed during the conduct of the review will be included in the published article and/or its Supporting Information.
