# Supplementary material for: Effectiveness of Extended Reality‐Based Cardiopulmonary Resuscitation Training for Healthcare Students: A Protocol for a Systematic Review and Meta‐Analysis
Source: Nurs Open. 2026 Jul 29;13(8):e70721. doi: 10.1002/nop2.70721 (PMC13420318; doi:10.1002/nop2.70721)
Supplement: Supplementary file 1 — Table S1: Eligibility criteria for the systematic review. [file NOP2-13-e70721-s001.docx]

**Supplementary Table 1**. Eligibility criteria for the systematic review.

| **PICO Element** | **Definition** | **Examples** |
| --- | --- | --- |
| **Population** | Undergraduate healthcare students | Nursing, medical, midwifery, or other undergraduate healthcare students.  Excludes laypersons, licensed professionals, or post-graduate trainees. |
| **Intervention** | XR-based CPR training | Training or simulation interventions using Extended Reality (XR) technologies, including:  • Augmented Reality (AR)  • Virtual Reality (VR)  • Mixed Reality (MR).  XR must be the primary training method. |
| **Comparison** | Conventional CPR training | This includes instructor-led training, manikin-based simulation without XR, classroom-based instruction, or other standard methods. |
| **Primary Outcome** | CPR Quality | Quantifiable measures of CPR performance, including:  • Chest compression fraction (%)  • Compression rate (per minute)  • Compression depth (mm)  • Avoidance of excessive ventilation (%) |
| **Secondary Outcomes** | Technical and non-technical skills | Technical: skills acquisition or retention;  Non-technical: confidence, communication, leadership, decision-making.  Outcomes must be reported in a quantifiable format suitable for meta-analysis (e.g., mean, SD, % correct). |
| **Study Design** | Randomized controlled trials (RCTs) and quasi-experimental studies. | Excludes observational studies, case reports, reviews, and abstracts without full data. |
